# Remote Monitoring and Virtual Appointments for the Assessment and Management of Depression via the Co-HIVE Model of Care: A Qualitative Descriptive Study of Patient Experiences

**DOI:** 10.3390/healthcare12202084

**Published:** 2024-10-18

**Authors:** Aleesha Thompson, Drianca Naidoo, Eliza Becker, Kevin M. Trentino, Dharjinder Rooprai, Kenneth Lee

**Affiliations:** 1 Community and Virtual Care Innovation, East Metropolitan Health Service, Perth 6000, Australia; 2 Armadale Kalamunda Health Service, Armadale 6992, Australia; 3 School of Allied Health, The University of Western Australia, Perth 6009, Australia

**Keywords:** remote patient monitoring, virtual care, virtual appointments, telehealth, digital health, depression, mental health, community care, qualitative, patient experience

## Abstract

**Objective:** This qualitative study sought to explore patient experiences with technologies used in the Community Health in a Virtual Environment (Co-HIVE) pilot trial. Technology is becoming increasingly prevalent in mental healthcare, and user acceptance is critical for successful adoption and therefore clinical impact. The Co-HIVE pilot trialled a model of care whereby community-dwelling patients with symptoms of depression utilised virtual appointments and remote monitoring for the assessment and management of their condition, as an adjunct to routine care. **Methods:** Using a qualitative descriptive design, participants for this study were patients with symptoms of moderate to severe depression (based on the 9-item Patient Health Questionnaire, PHQ-9), who had completed the Co-HIVE pilot. Data was collected via semi-structured interviews that were audio-recorded, transcribed clean-verbatim, and thematically analysed using the Framework Method. **Results:** Ten participants completed the semi-structured interviews. Participants reported experiencing more personalised care, improved health knowledge and understanding, and greater self-care, enabled by the remote monitoring technology. Additionally, participants reported virtual appointments supported the clinician–patient relationship and improved access to mental health services. **Conclusions:** This experience of participants with the Co-HIVE pilot indicates there is a degree of acceptance of health technologies for use with community mental healthcare. This acceptance demonstrates opportunities to innovate existing mental health services by leveraging technology.

## 1. Introduction

Health service delivery has rapidly evolved in recent times with the increased availability and utilisation of various health technologies. Health technology typically refers to the use of information and communication technology in the context of health and wellbeing, with common elements including telehealth, wearable devices, and mobile health [1,2]. Telehealth typically refers to the use of computers and mobile devices for audio-visual calls to provide clinical care remotely, addressing a number of barriers that prevent patients from attending health services in person [3,4,5]. Wearable devices include wearable technology with built-in sensors that measure and record users’ physiological health data, such as heart rate, temperature, and respiration rate, as well as health behaviours such as physical activity and sleep [6,7]. Mobile health refers to the use of mobile technologies for healthcare, for example, smartphone applications (apps) to deliver health questionnaires for users to complete [1,8,9].

Collectively, existing evidence demonstrates the benefits of health technologies to enhance the quality of clinical care for physical health conditions by improving access to services and enabling personalised interventions based on the individual’s own health data [10]. Comparatively, the uptake of health technologies by mental health services has been slow, with usage only increasing in light of the COVID-19 pandemic [9,11,12,13]. The slower uptake of technology in mental healthcare is likely multifactorial, but can include the perceptions of the negative impact technology has on mental health, that remote or virtually delivered care is less effective than in-person care, or that patients lack the level of digital literacy necessary to use technology safely and effectively [14,15,16]. Despite these perceptions, emerging evidence suggests there are clinical benefits for incorporating technology within mental healthcare, alongside indications that individuals with mental health conditions use technology at similar rates to the general population [17,18,19,20].

Mental health conditions affect more than 301 million people globally, and in Australia in 2020–2021, an estimated five million people experienced a mental health condition [21,22,23]. With the increasing prevalence of mental health conditions comes added health, social, and economic burden not only in Australia but worldwide [24,25]. Similar to the management of physical health conditions, health technologies can provide scalable, affordable, and accessible solutions to improve mental healthcare and effectively manage burden on an individual and service level [9,26,27]. When used clinically, mobile health apps used by patients to self-report on symptoms between service interactions can help to reduce recall bias and inaccuracies during clinical appointments, a common challenge for individuals experiencing mental health symptoms [8,26,28,29]. Additionally, wearable devices such as smartwatches have known benefits in supporting the management of physical health conditions through the capture of physiological health data [1,6]. For individuals experiencing mental health conditions, day-to-day physiological monitoring using wearable devices can provide useful insights into health behaviours, with future advancements having the potential to support the early detection of mental health deterioration [9]. Combining a variety of technologies to capture physiological and psychological health data for mental healthcare enables clinicians to remotely monitor the overall health of their patients and guide holistic clinical interventions tailored to the individual [8,9,28].

To harness health technology, the East Metropolitan Health Service (EMHS), in Perth, Western Australia, received federal grant funding to pilot the Community Health in a Virtual Environment (Co-HIVE) model of care, a research project trialling the use of health technologies for outpatient mental healthcare. The Co-HIVE model of care stemmed from the renowned Health In a Virtual Environment (HIVE) programme, which uses remote patient monitoring and artificial intelligence to detect signs of clinical deterioration in patients within hospitals across the state, and support local clinical teams to deliver proactive and timely interventions [30]. The Co-HIVE model of care was evaluated as a pilot Randomised Controlled Trial (RCT). The model of care utilised technology for remote physical and psychological health monitoring and virtual appointments for individuals with symptoms of depression receiving outpatient mental healthcare in the EMHS catchment. During the pilot, participants were randomly allocated to one of three study groups receiving routine outpatient mental healthcare, with interventions consisting of a smartphone application and smartwatch device for remote health monitoring, along with telehealth appointments for virtual check-ins and/or virtual health coaching (Table 1). Further details of the interventions used in the pilot RCT are described in Appendix A. During the pilot, Co-HIVE clinicians assessed these participants using standardised mental health outcome measures, completed at fortnightly virtual appointments, referred to as ‘virtual check-ins’. The pilot RCT primarily investigated the acceptability, feasibility and efficacy of the Co-HIVE model of care, with these findings to be published elsewhere.

With the advancements in health technologies, alongside shifting expectations for patients to actively participate in their care, it is essential to explore the experiences of users engaging with these new technologies. Having a greater understanding of patient experiences is critical to effectively design new services or improve existing services, particularly where a lack of technology acceptance and service engagement can pose significant challenges for implementing changes [31,32]. The present study aimed to qualitatively explore patient experiences with health technologies through the Co-HIVE model of care, as an adjunct to outpatient mental healthcare. Specifically, we sought to explore the experience of patients who were participants from the Intervention and Intervention Plus study groups, following the Co-HIVE pilot (Table 1).

## 2. Materials and Methods

### 2.1. Participants

After completion of the Co-HIVE pilot RCT, participants from the two intervention groups, in which the technology was utilised, were eligible for invitation to participate in the present study. These participants were contacted at completion of the pilot and invited to share feedback on their experience with the Co-HIVE service, and utilising the associated technology, in a semi-structured interview session. Table 2 outlines the overall inclusion criteria for participation in the Co-HIVE pilot, also applicable to this study. Informed consent was obtained from all participants who agreed to share their experience and feedback in semi-structured interviews for the present study.

### 2.2. Procedure

The present paper is a qualitative descriptive study utilising semi-structured interviews following the conclusion of the Co-HIVE pilot. Semi-structured interviews were either solely conducted by a member of the research team, AT, a health professional, or co-facilitated with a lived experience mental health consultant. All semi-structured interviews were conducted by online audio-visual calls using the Microsoft Teams application (‘Classic’ version 1.6.0011166) and recorded using the applications’ recording function.

For the semi-structured interviews, an interview guide was developed based on the Technology Acceptance Model, to understand user perceptions around ease of use, usefulness and satisfaction [33]. The interview guide used open-ended questions, with standardised prompts, relating to participant experiences using the remote monitoring technology and telehealth platform for virtual check-ins and/or virtual health coaching clinical appointments (Supplementary Materials File S1). In addition to user perceptions, interview questions also explored the benefits and limitations of the Co-HIVE service model and technology used, as well as overall satisfaction and recommendations for service improvement. Interviews were conducted until data saturation was achieved, which was defined as the point where no new themes were identified, as determined independently by two members of the research team.

To enable transparent reporting, the present study follows the Consolidated Criteria for Reporting Qualitative Research (COREQ) [34].

### 2.3. Analysis and Quality Assurance

For this analysis, an inductive coding approach to thematic analysis was implemented using the Framework Method [35]. This systematic approach involves seven steps: (1) transcription, (2) familiarisation with the data, (3) coding key information relevant to the research question, (4) developing an analytical framework collaboratively, (5) applying the framework, (6) categorising the data, and (7) interpreting the findings [35]. Semi-structured interviews were audio-recorded and manually transcribed clean-verbatim by AT and checked for accuracy against the recordings. For familiarisation, interview transcripts were reviewed and de-identified by AT, and imported to NVivo 14 (Windows version 14.23.2) for analysis.

Two researchers, AT and DN, independently coded one transcript, and then met to compare and discuss preliminary codes and categories, refining the framework iteratively. This framework was independently applied by both researchers to subsequent transcripts, with updates made when new codes emerged. Where coding decisions differed, KL, an experienced qualitative researcher on the research team, was consulted for adjudication. Once the final analytical framework was established, AT applied it to all transcripts for interpretation. To ensure credibility, DN also applied the framework independently to approximately 50% of the transcripts. All decision-making and changes were documented in an audit trail for confirmability and dependability.

## 3. Results

### 3.1. Overview

**Figure 1:**
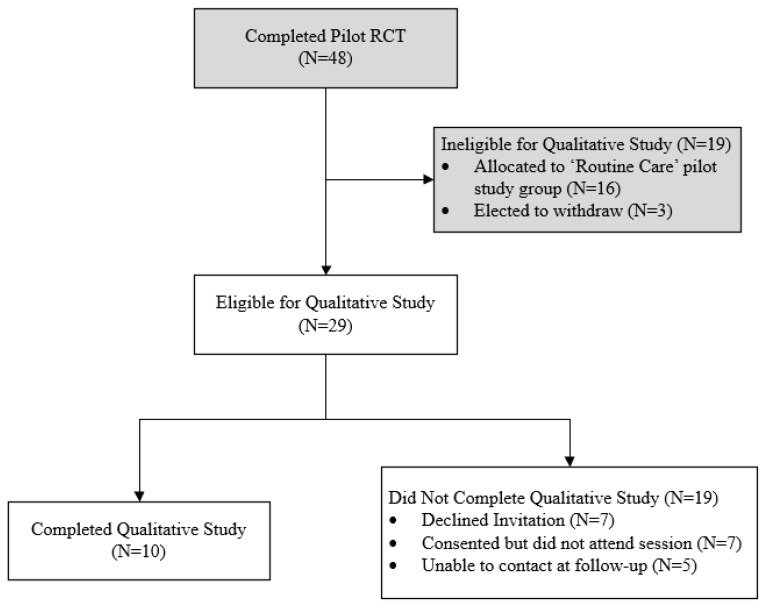
Flow of participants.

At completion of the Co-HIVE pilot, out of 48 participants, 29 participants were eligible for the present study and invited to complete semi-structured interviews to share their experience. Out of 29 eligible participants from the pilot, 19 did not participate in this study due to the following reasons: declined invitation (N = 7), agreed but did not attend their allocated session (N = 7), or were unable to be contacted at follow-up (N = 5). Ten participants completed the semi-structured interviews and therefore completed this study. The flow of participants is demonstrated in Figure 1. Participant demographics are outlined in Table 3. All semi-structured interviews were completed with participants individually and facilitated by AT, with one semi-structured interview co-facilitated by a lived experience mental health consultant. Semi-structured interviews were between 14 and 27 min (mean average 17.4 min). Data saturation was achieved on analysis of the fifth transcript. The following sections summarise the key findings from the present study, separated into manifest-level and latent-level themes. 

### 3.2. Manifest-Level Themes

The five manifest-level themes identified were Personalised Care with Health Data, Improved Access to Mental Healthcare, Supportive Therapeutic Relationships, Self-Care and Responsibility, and Health Knowledge and Understanding. Manifest-level themes refer to the directly observable content in the words or phrases used by participants, without exploring deeper meaning or relationships. These themes were common across both Intervention and Intervention Plus participants. Participant numbering is based on the pilot trial.

#### 3.2.1. Personalised Care with Health Data

Participants across both study groups reported the ability to track and visualise their health and progress over time was a positive and largely innovative concept, enabled by the remote monitoring technology and virtual appointments.

One participant commented on the novelty of capturing and visualising their personal physical and psychological health data for use as part of their mental healthcare.

*“It was really good to see that I think cause it just shows you in a visual form on your progress. And I think that was, you know, something new. I’ve had treatment for a very, very long time, and that was something new that hadn’t seen before and that, you know, it was a good. It was a feel-good thing for me because there was a lot of areas where I saw improvement.”*

—(P22, female, 30, Intervention Plus).

Participants described how capturing and visualising their own health data provided them with objective validation relating specifically to their mental health progress, which also prompted self-reflection.

*“It was actually really nice cause before, it was kind of like people would say I was or it seemed like I was getting better, but I didn’t feel like I was feeling any better. But then seeing, after answering all the questions and then seeing it in a graph how it did improve, like how my results did improve it made me reflect on it a bit more and how I was actually feeling.”*

—(P40, female, 18, Intervention Plus).

Participants reported that having their personal health data readily available on their own smartphone was useful when engaging with other healthcare providers and external services, such as their psychologist or General Practitioner (GP), as this information was relevant and easily shared.

*“I use the different apps to just sort of track my physical and mental health cause I have like a lot of physical and mental issues. So using both of them, helps quite a lot just to be able to keep track of it and then have everything ready at hand, for like doctors and appointments and stuff.”*

—(P29, female, 20, Intervention).

Participants, particularly those from the Intervention Plus group, who received the additional intervention of virtual health coaching described feeling their care was more personal. This was a result of Co-HIVE clinicians using the remote monitoring health data to gain a better understanding of the participants’ personal circumstances and experiences, to inform the care and support they provided.

*“Yeah and it gives like mental health providers a bit more info on what, you know, their patients would be up to, if they’re actually okay or how they’re really going. You can tell a lot about how much someone, how much steps they take or how much sleep they have. You can get a kind of overview of where they’re at.”*

—(P14, female, 22, Intervention Plus).

#### 3.2.2. Access to Mental Healthcare

Overall, participants commonly reported that using the remote monitoring technology and virtual appointments during the Co-HIVE pilot was easy, flexible and comfortable whilst facilitating convenient access to mental health support. Participants described how using the technology allowed them to continue accessing mental healthcare without disrupting their personal responsibilities, travel and leisure time.

*“Having the VCI [virtual check-ins] has not stopped me from doing my trips away for a break. This program will be the best thing for those living rural and remote, knowing that there is someone keeping an eye out on them, also allowing them to keep working if they are on the farm, like now with harvest time being busy, and not having to spend hours away.”*

—(P13, female, 43, Intervention Plus).

Participants further commented on how the virtual appointments made receiving care much easier if they were feeling unwell. Participants further explained this would be especially helpful in the early stages of engaging in mental healthcare, in the event of physical illness or if it suited their personal preferences.

*“Yeah, like at the moment, like if I was sick, like I am at the moment and it’s so much easier just to do it like this [virtually]. It’s like I could just be laying in bed, you know, could just be talking to you.”*

—(P2, female, 21, Intervention).

*“It didn’t matter, but like I have a social phobia, so hopping online it was a lot better for me personally.”*

—(P34, female, 55, Intervention).

#### 3.2.3. Therapeutic Relationships

Participants reported on the positive interactions and comfort they experienced with the Co-HIVE clinicians delivering care through virtual appointments. Participants described being able to connect well with the clinician through the virtual modality and felt cared for by having these interactions regularly with a “friendly face” who showed interest in their progress.

*“It was good having someone wanting to know how you were doing on the other end of the phone, the video call. Someone who you don’t know, who’s concerned and interested. Everything was helping, yeah. Even if you did something monthly you know, just to jog someone’s memory. Some of whom are struggling, and they need that. A friendly face to talk to makes all the difference.”*

—(P27, male, 46, Intervention Plus).

Most participants involved in the pilot trial had virtual appointments with the same Co-HIVE clinician throughout their involvement, allowing supportive therapeutic relationships to develop.

*“I only worked with [Clinician 1], umm and like I said I found her to be a wonderful person, she made me feel very, very comfortable. We had such a good time. We had so many laughs. She just made it a lot, just a lot more comfortable, not like I’d normally be.”*

—(P34, female 55, Intervention).

In contrast, two participants reported issues relating to having varied Co-HIVE clinicians during their involvement, which affected the therapeutic relationship and their experience with the service. One of these participants stated this was an issue as it meant they had to repeat discussions on sensitive matters within their virtual appointment each time they engaged with a new clinician, creating discomfort, and therefore, it was not preferable.

*“It would help if it was always the same person. I know that’s not always possible but it was easier. Like [Clinician 1] for example, because I was used to [Clinician 1], then one day it wasn’t them which meant I had to go through a lot of stuff all over again. Repeat myself. With an accent like mine I don’t like doing it as it is.”*

—(P27, male, 46, Intervention Plus).

#### 3.2.4. Self-Care and Responsibility

Participants described instances of taking responsibility for their health in between clinical interactions, with the technology serving as a physical reminder or actively prompting them to implement positive behaviour changes or self-care strategies.

*“I went to kick the ball around with my son sort of thing, I would have a look [at the smartwatch] and understand. I got my steps up and my heart rate up, I do need to do that more often. I think you know, everyone talks about exercise and sleep and if you have something that’s reminding you all the time it gets you off your backside to get up and go for a walk.”*

—(P27, male, 46, Intervention Plus).

Participants reported the technology often supported them in implementing self-care strategies between clinic appointments, indicating they were adopting greater responsibility for their health. This element of responsibility was also related to increasing their awareness and understanding, which is explored further in theme 5 (Health Knowledge and Understanding).

*“It gave me a chance to have a look at what’s going on with me. I’ve been really bad with sleep for many years, so it did help a bit there, but like it just made me more aware of what was going on with my life and it gave me that chance to, even though I didn’t get very far with changing it, [it was] making me more comfortable, making me make sure I move more. The whole program just made me a lot more aware than I’ve ever been before.”*

—(P34, female, 55, Intervention).

This theme of responsibility and self-care was also closely related to theme 1 (Personalised Care with Health Data), where participants reported that being able to visualise and monitor their data at any time allowed them to see changes in their health, in particular, the effects of regularly implementing self-care strategies or positive behaviour changes on their psychological health. This gave participants a sense of achievement after seeing improvements trending in their data over time.

*“I’m very visual, so if I see those numbers I can say, OK, you know, I’ve gotta improve my sleep or do my exercise. And also, you feel proud, you know, when it’s a week where you’ve done really, really good and you talk about your steps and say, yeah, you did this amount of steps. It’s, yeah, I think it’s a really, it’s a positive thing.”*

—(P22, female, 30, Intervention Plus).

#### 3.2.5. Health Knowledge and Understanding

Participants described gaining increased awareness and understanding of their physiological and psychological health, which was attributed to the use of the remote monitoring technology and virtual appointments.

*“You can see your heart rate elevate and you can sort of understand when you’re in distress and it does, like it helps with your sleep tracking quite a lot. The physical side of stuff like being able to be active is quite a big thing in mental health, so it was good to be able to have that tracked.”*

—(P29, female, 20, Intervention).

Participants began to identify changes and patterns in their health data and how these impacted their psychological symptoms or feelings, both positively and negatively.

*“It was good to be able to see in numbers that there are physical symptoms that are probably causing a lot of the, you know, the upset and sadness and other symptoms of depression or anxiety. So, I think that was really good to monitor. And because you do see that the better sleep, you know the more you can think, you know the clearer you can think, so it was good to see that. And then it posed a question. You know, how much is physically related and how much is mental to what I was going through, and I think that wasn’t ever measured in any other way before.”*

—(P22, female, 30, Intervention Plus).

Participants described learning more about their overall health, including receiving support to interpret their health data with the virtual health coaching, as well as guidance to engage with other healthcare providers where relevant.

*“I like knowing all the stuff that’s going on with my blood pressure, my sleep, all that was interesting cause like I’ve been referred to a sleep specialist now, so I’ll know a lot more, that gave me more data for that.”*

—(P43, male, 53, Intervention Plus).

### 3.3. Latent-Level Themes

These manifest-level themes were found to be underpinned by two broader, latent-level themes: (1) health data and (2) virtual healthcare. Unlike manifest-level themes, latent-level themes are a deeper exploration of the underlying meaning, patterns or relationships not immediately obvious in the content of the words or phrases used by participants. Firstly, the concept of capturing health data via technology was found to be the basis for three themes: Personalised Care with Health Data, Health Knowledge and Understanding, and Self-Care and Responsibility. Participants reported experiencing personalised healthcare; this was attributed to the Co-HIVE clinicians incorporating the individuals’ health data captured via the technology to inform decision-making and tailor interventions. In addition, participants reported that visualising and monitoring their health data provided an opportunity to gain knowledge and understanding of their individual mental health circumstances, which further provided them with the opportunity to take responsibility and adopt an active role in their care.

*“It was really good to see that I think cause it just shows you in a visual form on your own progress. And I think that was, you know, something new. I’ve had treatment for a very, very long time, and that was something new that I hadn’t seen before and, you know it was a feel-good thing for me because there was a lot of areas where I saw improvement.”*

—(P22, female, 30, Intervention Plus).

The second latent-level theme surrounded the concept of virtual healthcare, and unified themes of Access to Mental Healthcare and Therapeutic Relationships. Participants described the concept of accessing mental healthcare virtually mitigated a number of internal and external challenges that can commonly impact engagement, particularly for mental healthcare. Along with this, the virtual modality facilitated more regular and frequent interactions with clinicians, compared to routine care, leading participants to feel supported and able to establish effective therapeutic relationships.

*“Having someone I know that was a point of reference and knowing that they would be there in a couple of weeks’ time, sort of gave me the confidence to pick myself up and try and make it through that fortnight because I knew that there was gonna be someone there at the end of that fortnight.”*

—(P34, female, 55, Intervention).

## 4. Discussion

### 4.1. Primary Findings

The present study is the first study to provide qualitative insight into participants’ experiences using technology for remote monitoring of both psychological and physical health, alongside virtual appointments, for the assessment and management of depressive symptoms through community care. Participants of the present study reported positive experiences and acceptance of the technology as part of their mental healthcare delivered via the Co-HIVE service. These participant experiences highlight the pivotal role health data play in enabling clinicians to provide care that is more relevant to the individual, improves their understanding and supports behaviour changes. Participants reported that the use of virtual appointments for mental healthcare enabled accessibility to mental health support, without compromising the clinician–patient relationship, potentially even enhancing it.

#### 4.1.1. Health Data

In this study, we identified that remote monitoring technology enabled participants to experience personalised mental healthcare. The timely capture of relevant health data through the use of health technologies supports clinicians to make informed decisions for care, based on the individual [6,8,9]. Participants in the present study reported feeling personally supported and that their clinicians had a better understanding of their individual circumstances. This is thought to be attributed to the technology used to capture regular psychological and physiological health data of each participant over weeks and months. Co-HIVE clinicians could use these data to monitor acute health changes and progress over time, and guide decision-making on relevant interventions and management for the individual. In routine care, assessments rely heavily on patients retrospectively self-reporting to clinicians their symptoms and experiences over the weeks or months prior [28]. This can be challenging for both the clinician and patient, particularly if they are experiencing common symptoms associated with several mental health conditions, such as poor concentration, mood fluctuations, hopelessness, low energy and lack of sleep [28,35]. The absence of sufficient and/or accurate information from the patient limits clinicians’ ability to make informed decisions and therefore deliver care tailored to the individual [9,14,36]. Thus, the findings from the present study suggest remote monitoring technologies applied to mental healthcare provide clinicians with reliable and timely information, facilitating more personalised care.

Along with experiencing more personalised care, participants reported gaining awareness and understanding of their overall health, which in turn motivated them to enact behaviour or lifestyle changes. Empowering patients with knowledge and understanding is known to facilitate better self-management, inform decision-making, and improve health outcomes and service satisfaction [37]. The experience of participants in this study is an insightful finding considering the higher prevalence of poor physical health and associated health outcomes disproportionately affecting individuals experiencing mental health conditions, in particular depression [38,39,40,41]. The technology used in the pilot provided participants with instant access to view their own health data in a patient-friendly format, allowing a form of self-monitoring. Being able to self-monitor their health provided additional learning opportunities for these participants, independent of service interactions or clinic appointments. Whilst not all participants received virtual health coaching to support the interpretation of their health data, we found that the theme of increased awareness prevailed irrespective of whether participants received this component of the intervention. In addition, participants often reported on the benefit of using their health information with other healthcare providers involved in their care. This finding suggests that simply having information available directly to the participant at any point in time enabled participants to adopt an active role in their mental healthcare and implement self-care strategies or positive behaviour changes, whilst also having the potential to facilitate care across healthcare providers.

Healthcare that is solely reliant on short, infrequent and sometimes difficult-to-access, face-to-face appointments limits the availability and access that patients have to their own health information [42]. Having the opportunity to freely access information empowers patients by giving them the opportunity to view and use this information with other healthcare providers, as well as implement health behaviour changes in their daily lives. Previous studies support the use of technology in mental healthcare, as it can complement in-person appointments, through the added benefit of visibility of their health information between sessions, which would otherwise not be available [12,43]. Our findings suggest that incorporating self-monitoring, as part of a multi-modal mental healthcare approach, may empower patients by enhancing awareness, understanding and responsibility. This approach could be particularly beneficial during transitions from high-acuity to community-based care, as it may assist in gradually reducing dependence on clinical service support. The unique feature of capturing and monitoring physical health data in this population has further potential to improve the physical health outcomes of individuals living with mental health conditions.

#### 4.1.2. Virtual Healthcare

Participants in the present study described feeling able to develop supportive therapeutic relationships through virtual appointments. The development of supportive therapeutic relationships could be predominantly attributed to personalised and continuity of care but appeared to be enhanced by features unique to the virtual healthcare modality. Participants often reported being eager to attend their virtual appointments, as it meant they could attend from home or another location of their choice, allowing for increased privacy, comfort and security. Previous studies have found that delivering care in an environment where patients feel comfortable and at ease to share information, such as the experience with virtual appointments, supports the development of effective therapeutic relationships [44]. However, care delivered through virtual or online appointments may be perceived as contributing to social avoidance, and less conducive to developing these relationships typically established through in-person sessions [9,10,45]. Furthermore, user acceptance, indicated by engagement with health technologies, has been challenging to determine in previous studies involving technology in the treatment and management of depression [31,32]. Common barriers to engagement with health technologies have been found to relate to the presence and severity of depressive symptoms, including low mood, lack of motivation, and poor memory and insight [15]. The findings from this study challenge this, where participants were accepting of the technology when experiencing supportive therapeutic relationships while receiving care virtually.

Along with providing an environment conducive to developing supportive therapeutic relationships, the convenience and flexibility of virtual care improved participants’ experience accessing mental health services. Participants reported positively on being able to flexibly access care via virtual appointments from their home or work or whilst travelling, negating the requirement to attend in-person appointments. This allowed participants to continue to receive care with minimal disruption to other aspects of their life, or it offered them a discreet option if preferred. Offering virtual care reduced some of the common challenges patients experience when accessing mental healthcare, such as mental health stigma, associated psychological symptoms or geographical barriers [15,46,47,48,49,50]. Although the present study did not involve participants living in rural and remote areas, many participants commented on how beneficial virtual services would be in these areas, where mental health services are limited or geographically dispersed [49]. Participants in the study commented on the convenience of virtual appointments which allowed them to commence or continue to access services even when their symptoms may have been severe or had acutely deteriorated. Previous studies suggest that common symptoms such as low mood, lack of motivation and fatigue can make the effort of attending clinic appointments significantly challenging for people experiencing mental illness [15,28,51]. In this study, virtual appointments were found to offer participants an easy, low-effort option to receive care and continue engaging with mental health support. As health technologies become more common, virtual care will likely be a valuable option to offer to suit the needs and preferences of individuals and support their access to and engagement with mental healthcare.

### 4.2. Limitations

Although our findings suggest positive experiences and support the emerging acceptance of technology use in mental healthcare, it has a number of limitations. For example, the nature of qualitative research is based on the researchers’ interpretation of subjective information. To improve rigour, we kept an audit trail of discussions and decision-making throughout the analysis process, used a codebook, and employed analyst triangulation. Using a pre-defined template, our audit trail was a log of when (date and time stamps) and why decisions were made in our approach to coding/theming. In a similar manner, we used a pre-defined template to list all the codes we used in our qualitative analysis. We used this to create a structured system to label and categorise our data. Our codebook included codes and descriptive examples for each code. Our analysis triangulation involved three investigators independently interpreting the data and developing a collaborative framework.

Our use of a convenience sampling method may limit the generalisability of these findings. However, it is to be noted that generalisability is not the intent of qualitative research, and our decision to include demographic descriptors alongside participant quotes serves to transparently provide readers with information as to who our findings could potentially apply to. Furthermore, despite a convenience sample, the demographics of our sample of participants are comparable to the demographics of the typical outpatient mental health service users in metropolitan Australia, with similar representations of Aboriginal and/or Torres Strait Islander people, as well as a proportional presence of eight participants identifying as female, inclusive of transgender individuals who identify as female [22].

### 4.3. Implications for Policymakers and Future Research

Participants in our study reported positive experiences and acceptance of digital technology as part of their mental healthcare. Despite this, we recognise there are extensive challenges and pitfalls associated with utilising digital technology within mental healthcare. Some of the relevant key challenges include the ‘digital divide’, limited applicability or suitability, and concerns around data privacy and security [52,53].

The varying level of skills necessary to use technology, as well as means to access it, is often described as the ‘digital divide’. Research presents conflicting results on the digital literacy levels of patients with mental health conditions [20]. It has been found that people with severe mental ill health have lower levels of digital health literacy, and therefore are less likely to engage with technology [54]. The digital divide may be more prevalent in patients with mental health conditions, who are more likely to encounter lower socio-economic status [54,55]. For example, patients receiving community mental health rehabilitation were found to be less likely to use mobile phones, computers and the internet, compared with the general population [53]. This was reported to be mainly due to a lack of access to the technology, or understanding and skills necessary to use it [53]. Furthermore, whilst the use of technology in older adult populations is increasing, a digital divide remains, with older adults less likely to use technology, finding it more challenging [56]. Therefore, the use of technologies within mental healthcare is not likely to be suitable or applicable to all mental health conditions. This digital divide applies not only to patients but also to clinicians delivering interventions that rely on such technologies.

Another key obstacle to the implementation of such technology within mental healthcare is the common concerns relating to the confidentiality and privacy of sensitive patient health data [52]. This challenge also interplays with consumer trust in health services implementing such technologies. Trusting that health technology is safe and secure is a significant factor for user acceptance. Without trust and acceptance of technology and the health systems that manage it, patients, as well as clinicians, are less likely to use it, mitigating any potential benefits it may offer [57,58].

Whilst these are only a small portion of the challenges surrounding technology use within mental healthcare, addressing these obstacles will require collaboration from a variety of stakeholders including patients, health professionals, digital health experts, researchers and policymakers.

We suggest future research explore the use of health technologies to support care for other mental health conditions. This study primarily explored the experience of low-risk participants with symptoms of moderate to severe depression. Exploring the use of health technologies with other mental health conditions would be useful to provide insight into broader patient experiences incorporating technology in mental healthcare. Additionally, exploring the use of these technologies with patients of different age groups, such as those younger than 18 or older than 65, may reveal whether age impacts their experience.

## 5. Conclusions

Remote monitoring and virtual appointments, when applied to mental healthcare, can broaden the reach of routine care and facilitate the delivery of more personalised, timely care. By improving the reach of mental healthcare, this technology addresses some of the barriers that prevent individuals from seeking or continuing to engage in care. In addition to supporting service utilisation and engagement, incorporating technology also provides an opportunity for patients to adopt an active role in their healthcare, by empowering them with information and supporting greater understanding. As technology and virtual modalities become an increasingly common part of everyday life, patients are becoming more accepting of the integration in healthcare and experiencing various benefits. Leveraging health technology to innovate mental healthcare delivery will be a crucial direction to explore, as the demand for mental health services increasingly exceeds capacity. Understanding the needs, expectations and preferences of patients is critical for mental health services seeking to incorporating health technologies and achieve the desired clinical and broader health system benefits. Further research is recommended to investigate the clinical outcomes among patients receiving mental healthcare that incorporates health technologies or virtual services.

## Figures and Tables

**Table 1:** Study groups for the Co-HIVE pilot RCT.

Study Group	Components of Intervention		
	Routine Care ^1^	Remote Monitoring ^2^	Virtual Health Coaching ^3^
Control	✔		
Intervention	✔	✔	
Intervention Plus	✔	✔	✔

^1^ Routine outpatient mental healthcare typically delivered through in-person appointments with a mental health clinician, (Appendix A.1). ^2^ Remote monitoring of participant physiological and psychological health data captured via a smartwatch (Fitbit Charge 5) and smartphone application (Mentegram) delivering standardised mental health outcome measures. Data from both devices were monitored remotely by Co-HIVE clinicians based in a tertiary hospital (Appendix A.2). ^3^ Virtual health coaching involved clinical intervention and support delivered by Co-HIVE clinicians via the telehealth platform HealthDirect (Appendix A.3).

**Table 2:** Participant inclusion criteria.

Inclusion Criteria
Receiving outpatient mental healthcare through an EMHS Community Mental Health Service (CMHS) Aged 18 to 65 years (inclusive) Moderate to severe depression based on Patient Health Questionnaire ^4,5^ (PHQ-9) score > 10 No, low, or moderate suicide and/or violence risk No active psychosis Allocated to either Intervention or Intervention Plus study groups in the Co-HIVE pilot RCT ^6^

^4^ Based on clinical assessment by referring CMHS clinician. ^5^ Adapted PHQ-9 used if participant identified as Aboriginal and/or Torres Strait Islander. ^6^ Criteria relevant only for present study.

**Table 3:** Participant demographics.

Participant Demographics	
Median age, years (Q1–Q3)	26 (20–50)
Aboriginal and/or Torres Strait Islander, n (%)	1 (1.0)
Median PHQ-9 score at baseline (Q1–Q3)	19.5 (13–23)
Gender Identity, n (%)	
Male	2 (20.0)
Female	7 (70.0)
Transgender female	1 (10.0)
Transgender male	0 (0.0)
Non-binary/other	0 (0.0)
Pilot RCT Study Groups, n (%)	
Intervention	5 (50)
Intervention Plus	5 (50)

## Data Availability

The datasets presented in this article are not readily available due to privacy restrictions. Requests to access datasets can be directed to the corresponding authors.

## Supplementary Materials

The following supporting information can be downloaded at https://www.mdpi.com/article/10.3390/healthcare12202084/s1: File S1: Semi-structured interview questions.

## Appendix A

The following section provides supplementary information regarding ‘Routine Care’, and the interventions of ‘Remote Monitoring’ and ‘Virtual Health Coaching’ used for the Co-HIVE pilot RCT.

### Appendix A.1. Routine Care

Routine care consisted of routine outpatient community mental healthcare, delivered by Community Mental Health Services (CMHS) within the East Metropolitan Health Service. This included the allocation of a Case Manager to provide 10 to 12 weeks of mental health clinical assessment and support, as well as care coordination with a multi-disciplinary team including psychiatrists, nurses and allied health professionals. These service interactions are routinely delivered through face-to-face appointments with consumers at the relevant CMHS site, their home or in the community. Depending on their individual needs, consumers may have received various clinical interventions including psychosocial intervention, crisis resolution, commencement and review of medications, admission diversion and facilitated discharges. As reflected in routine practice, the clinical care received by those in the control group of the pilot RCT varied according to individual needs but excluded any interactions using remote monitoring technology or virtual appointments.

### Appendix A.2. Remote Monitoring

Remote monitoring interventions used in the pilot RCT consisted of two components of technology that captured and recorded participants’ physical and psychological health data. The first component of monitoring technology consisted of a smartwatch (Fitbit Charge 5) worn by participants continuously to capture physical health data, including heart rate, activity (step count) and sleep (minutes). The second component of monitoring technology consisted of a smartphone application (Mentegram), used to electronically deliver standardised outcome measures and questionnaires to participants for them to complete at scheduled intervals (e.g., daily, weekly, fortnightly). These outcome measure and questionnaires aimed to capture the psychological health data of participants. Standardised outcome measures delivered via the application included the Patient Health Questionnaire (PHQ-9), Hamilton Depression Rating Scale (HDRS), Beck Depression Inventory (BDI-II), Depression Anxiety Stress Scale (DASS-21) and the Columbia-Suicide Severity Rating Scale (C-SSRS). Where participants identified as Aboriginal and/or Torres Strait Islander, the Adapted Patient Health Questionnaire (aPHQ-9) was used. Questionnaires delivered via the application captured general health information from participants, for example, relating to medication side effects, sleep quality and mood. Health data captured through these two components of monitoring technology were visible to Co-HIVE clinicians based remotely at Royal Perth Hospital for monitoring. When data indicated a participant was experiencing potential physical or psychological health deterioration, the Co-HIVE clinicians would contact the participant directly via text message, phone or audio-visual call to check in. In addition to direct contact with the participant, where appropriate, the Co-HIVE clinician would contact the participants’ relevant CMHS Case Manager for care collaboration. Participants in the ‘Intervention’ study group of the pilot RCT received this remote monitoring in addition to routine care.

### Appendix A.3. Virtual Health Coaching

For the pilot RCT, virtual health coaching consisted of clinical intervention and support delivered by Co-HIVE clinicians through virtual appointments. Virtual appointments were completed with the clinician and participant through a telehealth platform (HealthDirect). Virtual health coaching appointments were completed fortnightly with each participant receiving this intervention. Co-HIVE clinicians provided clinical care and support based on the participants’ health data captured by the remote monitoring technology. The aim of virtual health coaching was to support participants to interpret their personal health data and identify strategies to help improve areas of their health. For example, to support a participant to improve their sleep, Co-HIVE clinicians would suggest strategies for managing sleep hygiene or potential medication side effects. Participants in the ‘Intervention Plus’ study group of the pilot RCT received this virtual health coaching, in addition to remote monitoring and routine care.
