# Supplementary material for: Remote Monitoring and Virtual Appointments for the Assessment and Management of Depression via the Co-HIVE Model of Care: A Qualitative Descriptive Study of Patient Experiences
Source: Healthcare (Basel). 2024 Oct 18;12(20):2084. doi: 10.3390/healthcare12202084 (PMC11508023; doi:10.3390/healthcare12202084)
Supplement: Supplementary file 1 [file healthcare-12-02084-s001.zip › healthcare-3169870-supplementary.pdf]

Supplementary Materials

Attachment S1: Semi-Structured Interview Questions.

QUESTIONS	PROMPTS
1. A) How did you first hear about the Co-HIVE Service?<br>B) What was your experience regarding the initial Onboarding when commencing with Co-HIVE?	*A) Reception paperwork, Care Coordinator, Treating Doctor.*<br>*B) Sufficient information? Was the involvement agreement clear, did you understand the requirements of your involvement? Access to support?*
2. What was your experience with the Virtual Check-Ins?	*Positives, negatives what could change?*
3. What was your experience engaging in discussions with the clinician?	*Have you completed other healthcare related video calls? What do you like/ do not like?*
4. What was your experience with accessing and completing the Mentegram questionnaires?	*Positives, negatives, what could change?*<br>*Prompt for time commitment, did it change how or what you did in your daily routines?*
5. What motivated you to complete the tasks in the study?	*Prompt for completing daily/ weekly / fortnightly questionnaires, readiness for virtual check ins, use of the device.*
6. What, if anything, did you find good about your involvement experience in Co-HIVE?	*Did you experience any benefits?*<br>*Did your experience add value to your mental health care recovery?*<br>*Can you recommend how we could change or improve these aspects?*
7. What, if anything, did you find not good about the involvement experience in Co-HIVE?	*Were any issues or concerns addressed, if so, how?*<br>*Can you recommend how we could change or improve these aspects?*
8. How did the technology fit in with your mental healthcare?	*Could the technology support your mental healthcare? If so, how?*
9. Overall, how did you find the technology?	*Positive, negatives, what could change?*<br>*Were the expectations met?*
10. Would you recommend Co-HIVE to other mental health consumers and why?	
11. What impacts on your daily life did using the smartwatch device have?	*Prompts for time commitment, did it change how or what you did in your daily routines*
12. Did you experience any benefits in your physical health from using the smartwatch device?	*Facilitator to ensure that each participant responds*
13. Factoring in the lifestyle impacts, were there enough benefits to using the smartwatch device to make the impacts worthwhile?	*Prompt for health awareness/practices carrying over into day-to-day activity*
14. What, if anything, did you find good about the smartwatch you were given?	*Prompts to consider 'ease of use'*
15. What, if anything, did you find was not good about the smartwatch you were given?	*Facilitator to ensure that each participant responds,*<br>*Prompts for 'barriers of use'*
16. If you could change anything about the smartwatch, what would it be?	
17. How do you see the technology fitting in with your regular mental healthcare?	

18. Did your involvement with Co-HIVE prompt you to seek any further community service engagement i.e GP Care regarding sleep/lifestyle/heart care?	
19. Would you recommend the smartwatch to other mental health consumers and why?	
20. Overall, how did you find the device (Fitbit)?	
